# c-Met and PD-L1 on Circulating Exosomes as Diagnostic and Prognostic Markers for Pancreatic Cancer

**DOI:** 10.3390/ijms20133305

**Published:** 2019-07-05

**Authors:** Alexander Lux, Christoph Kahlert, Robert Grützmann, Christian Pilarsky

**Affiliations:** 1 Medizinische Klinik III, Universitätsklinikum Carl Gustav Carus Dresden, Fetscherstraße 74, 01307 Dresden, Germany; 2 Klinik und Poliklinik für Viszeral-, Thorax- und Gefäßchirurgie, Universitätsklinikum Carl Gustav Carus Dresden, Fetscherstraße 74, 01307 Dresden, Germany; 3 Department of Surgery, Universitätsklinikum Erlangen, Krankenhausstraße 12, 91054 Erlangen, Germany

**Keywords:** pancreatic cancer, PDAC, c-Met, PD-L1, exosomes, chronic pancreatitis, serous cyst adenoma

## Abstract

Exosomes are membrane vesicles which offer potential as blood derived biomarkers for malign tumors in clinical practice. Pancreatic cancer is counted among cancer diseases with the highest mortality. The present work seeks to assess whether pancreatic carcinomas release exosomes which express c-Met (proto-oncogene mesenchymal-epithelial transition factor) and PD-L1 (programmed cell death 1 ligand 1), and whether the detection of such expression in serum has diagnostic or prognostic meaning for the affected patients. Exosome isolation was performed on culture media of one benign pancreatic cell line and ten pancreatic carcinoma cell lines as well as on serum samples from 55 patients with pancreatic ductal adenocarcinoma (PDAC), 26 patients with chronic pancreatitis and 10 patients with benign serous cyst adenoma of the pancreas. Exosomes were bound to latex beads and stained with antibodies against c-Met or PD-L1. Analysis of fluorescence intensity was performed by flow cytometry. In terms of c-Met, the mean fluorescence intensity of PDAC-patients was significantly higher than the fluorescence intensity of the comparative patients with benign disease (*p* < 0.001). A diagnostic test based on c-Met resulted in a sensitivity of 70%, a specificity of 85% and a diagnostic odds ratio of 13:2. The specificity of the test can be further improved by combining it with the established tumor marker carbohydrate antigen 19-9 (CA 19-9). In addition, c-Met-positive patients showed a significantly shorter postoperative survival time (9.5 vs. 21.7 months, *p* < 0.001). In terms of PD-L1, no significant difference between fluorescence intensity of PDAC-patients and comparative patients was detectable. However, PD-L1-positive PDAC-patients also showed a significantly shorter postoperative survival time (7.8 vs. 17.2 months, *p* = 0.043). Thus, both markers can be considered as negative prognostic factors.

## 1. Introduction

According to data of the Robert Koch Institute, about 17,100 people in Germany developed a malignant neoplasia of the pancreas in 2014 [1]. Among the malignant neoplasias of the pancreas, pancreatic ductal adenocarcinoma (PDAC) is the most common histological subtype, accounting for more than 90% of the cases [2]. This malignancy is characterized by a particularly aggressive and rapidly infiltrative growth with a relatively long asymptomatic course. Therefore, the diagnosis is often made in advanced tumor stages. Due to this fact, pancreatic carcinoma has the lowest relative five-year survival rate among all common cancer types with 9% in men and 10% in women. It is also the fourth most common cause of cancer deaths in both genders in Germany [1].

Exosomes are naturally formed membrane vesicles with a diameter of about 40 to 100 nm which are distinguished by a typical, cup-shaped appearance in electron microscopy [3]. They belong to the superordinate group of extracellular vesicles (EV) which are surrounded by a lipid bilayer with anchored transmembrane proteins and contain soluble proteins as well as RNA. Since exosomes can be isolated from almost all body fluids, their potential use as biomarkers is obvious [4].

The receptor tyrosine kinase c-Met (proto-oncogene mesenchymal-epithelial transition factor), also known as hepatocyte growth factor receptor (HGFR), is the gene product of the proto-oncogene MET on chromosome 7 [5]. Key signaling cascades controlled by c-Met include the mitogen-activated protein kinase (MAPK) pathway, the phosphoinositide-3-kinase (PI3K) pathway, the signal transducers and activators of transcription (STAT) pathway as well as the NF-κB pathway (nuclear factor “Kappa Light Chain Enhancer” of activated B-Cells), which overall provide for cell proliferation, cell migration and apoptosis protection [6]. By this means, activation of c-Met leads to invasive growth of cell aggregates, which harbors the dangerous potential to contribute to the development of malignant tumors. Its expression is known to be upregulated in the majority of PDACs [7].

The transmembrane protein PD-L1 (programmed cell death 1 ligand 1); also known as B7-H1 (B7 homolog 1) or CD274, is a ligand of the PD-1 receptor (programmed cell death protein 1). PD-1 is called a “checkpoint” of the immune system because its interaction with PD-L1 is able to limit the specific immune response [8]. The physiological function of PD-1 is to prevent an excessive immune response and to assure the tolerance of harmless antigens and endogenous tissue. Tumor cells, however, seem to take advantage of this mechanism by expressing PD-L1 in order to evade immune control. Increased PD-L1 expression has been demonstrated in malignancies of the skin, brain, thyroid, esophagus, colorectal and, among others, the pancreas [9].

The present work attempts to evaluate whether pancreatic carcinoma cells produce exosomes expressing the surface proteins c-Met and PD-L1, and whether this expression may have diagnostic or prognostic meaning.

## 2. Results

We investigated the expression status of c-Met and PD-L1 on exosomes gained from culture media of one benign and ten malignant human pancreatic cell lines as well as from serum samples of patients with PDAC, chronic pancreatitis and serous cyst adenoma of the pancreas. Due to the limited amount of available serum only a part of the patients could be tested with both antibodies.

To achieve sufficiently large comparison groups, patients with SCA were tested only on c-Met and the primary irresectable patients were preferably tested with PD-L1. The remaining patient sera were randomly assigned to both antibodies. Table 1 and Table 2 provide an overview of gender distribution, mean age, TNM stages and treatment intentions of the total patient collective and the subgroups analyzed with c-Met and PD-L1.

**Figure 1:**
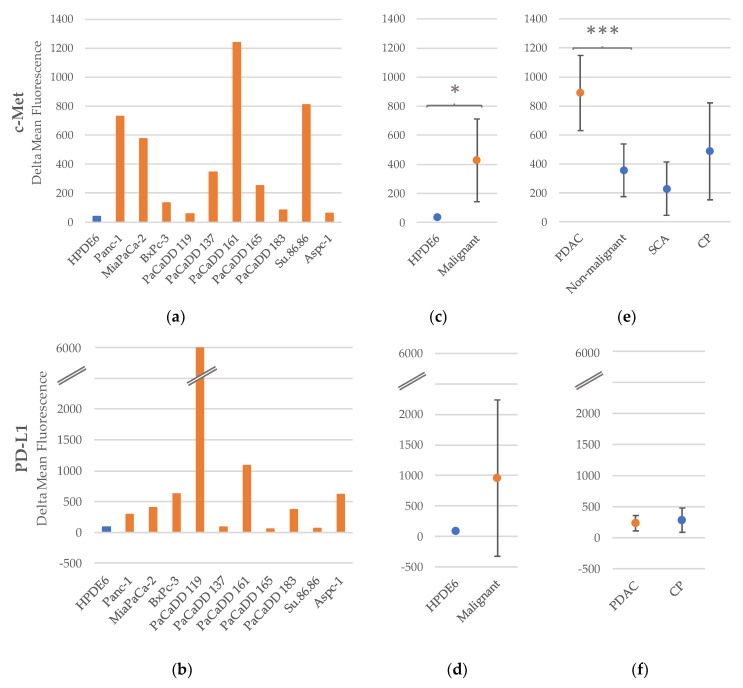
Fluorescence intensity of human pancreatic cell lines using the primary antibody anti-c-Met (**a**) and PD-L1 (**b**); (**c**) mean estimator and 95% confidence interval (CI) of malignant cell lines compared to benign human pancreatic duct epithelial cell line (HPDE) using c-Met (**c**) and PD-L1 (**d**); (**c**) mean estimators and 95% CI of the fluorescence intensities of various disease entities using c-Met (**e**) and PD-L1 (**f**); * significant at 5% level (*p* < 0.05); *** significant at 0.1% level (*p* < 0.001).

The benign cell line HPDE6 displays only a small difference between the positive and negative control using both antibodies c-Met and PD-L1. The mean fluorescence intensity of the malignant cell lines using anti-c-Met is 427.5 ± 286.0, which is significantly higher than the fluorescence of HPDE6 (*p* = 0.013) (Figure 1a,c). Using anti-PD-L1 on exosomes gained from cell culture media, the strong variance between the different malignant cell lines results in a broad 95% confidence interval (954.9 ± 1277.4). Thus, there is no significant difference to HPDE6 (Figure 1b,d).

Examining exosomes gained from patient sera, we found a significant difference between PDAC patients and patients with a non-malignant differential diagnosis using anti-c-Met (Figure 1e, Table 3). In case of anti-PD-L1, we found no significant difference between PDAC and chronic pancreatitis (CP) patients (Figure 1f, Table 4).

### 2.1. c-Met as a Diagnostic and Prognostic Marker

In order to analyze the expression of c-Met as a diagnostic test, a cut-off value is needed, which must be reached to consider a sample positive. This value is determined by means of a receiver operating characteristics (ROC) curve. Among the positive serum samples tested, patients who are actually suffering from pancreatic carcinoma are highly significantly more prevalent (*p* < 0.001 in the chi-squared homogeneity test). The resulting diagnostic test has a sensitivity of 70%, a specificity of 85% and a diagnostic odds ratio of 13:2. 

**Figure 2:**
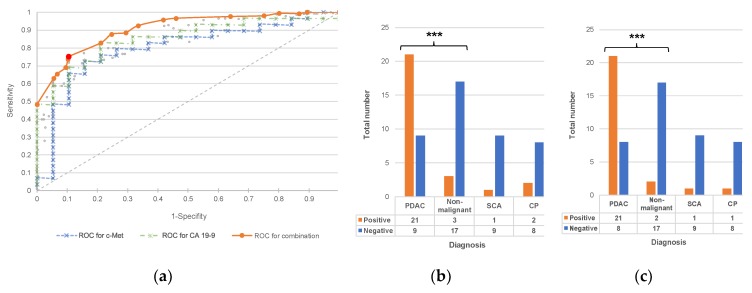
(**a**) Receiver operating characteristics curve of a diagnostic test based on c-Met, carbohydrate antigen 19-9 (CA 19-9) and the combination of both. (**b**) Contingency table of a test based on c-Met, using a cutoff value of >495. (**c**) Contingency table of a test based on the combination of c-Met and CA 19-9, using c-Met >543 or CA 19-9 >97 U/mL as cutoff. *** significant at 0.1% level (*p* < 0.001).

To improve the diagnostic test presented here, a cutoff optimization was also performed for the combination of c-Met and the clinically established biomarker carbohydrate antigen 19-9 (CA 19-9). In combination, a sensitivity of 72.4% is accompanied by an increased specificity of 89.5%. In the case of one patient with PDAC and one patient with CP there was no CA 19-9 value available; thus, they were not considered in this analysis (Figure 2, Table 5).

**Figure 3:**
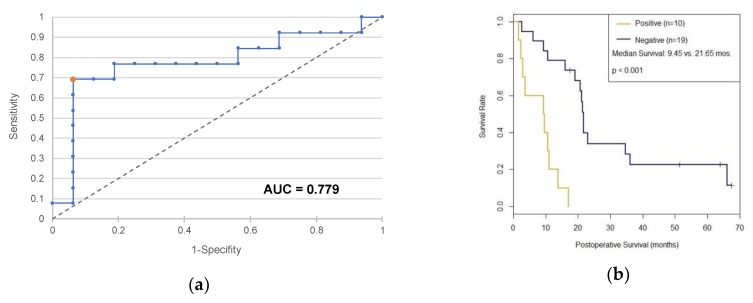
(**a**) ROC curve of a prognostic test based on c-Met. (**b**) Survival analysis according to Kaplan–Meier as a function of c-Met status using >990 as cutoff. Non-informative censors are marked as a dash.

By analogy with the cutoff determination for the diagnostic test, a ROC analysis was performed to examine the prognostic value of c-Met. For this purpose, the median survival time of all observed PDAC patients of 15.3 months was considered for separation between PDAC patients with long and short survival time (Figure 3a). In case of one patient, follow-up data were missing, so only 29 PDAC patients were considered in the subsequent analysis. A Kaplan–Meier survival time analysis was performed comparing patients with positive c-Met expression status and patients with negative c-Met expression status. The two resulting survival curves are shown in Figure 3b. The analysis reveals a median survival of 21.65 months [19.06-∞] for patients tested negative for c-Met and 9.45 months [2.83-∞] for positive patients. According to the log-rank test, this difference is statistically significant at the 0.1% level (*p* < 0.001).

### 2.2. PD-L1 as a Diagnostic and Prognostic Marker

**Figure 4:**
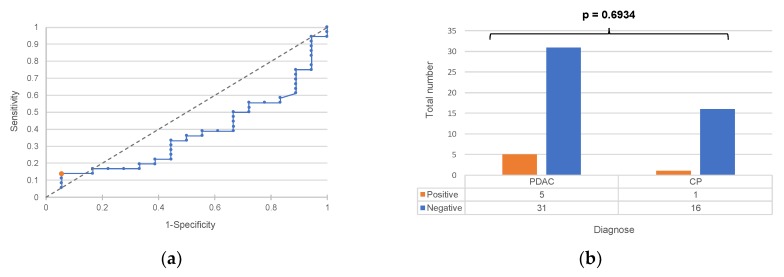
(**a**) ROC curve of a diagnostic test based on PD-L1. (**b**) Resulting contingency table of the test using a cutoff value of >512. *p*-value is based on a chi-squared homogeneity test.

The ROC curve in Figure 4a was used to determine the cutoff value for a diagnostic test based on PD-L1. The corresponding contingency table is shown in Figure 4b. The *p*-value based on a chi-squared homogeneity test is 0.3583; a significant difference between the two groups does therefore not exist. The resulting diagnostic test has a sensitivity of 14%, a specificity of 94% and a diagnostic odds ratio of 2:7.

**Figure 5:**
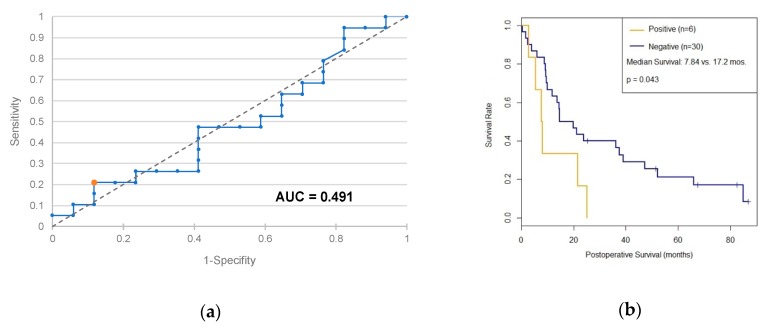
(**a**) ROC curve of a prognostic test based on PD-L1. (**b**) Survival analysis according to Kaplan–Meier as a function of PD-L1 status using >299 as cutoff. Non-informative censors are marked as a dash.

In order to analyze PD-L1 as a prognostic marker, a new cutoff optimization was performed to distinguish more precisely between long and short surviving patients (Figure 5a). A Kaplan–Meier survival time analysis was performed which differentiates between patients with positive and negative PD-L1 status. The resulting survival curves are shown in Figure 5b. The median postoperative survival of PD-L1 negative patients is 17.2 months (11.8-47.1); PD-L1-positive patients survived 7.84 months (5,32-∞). The *p*-value based on the log-rank test is 0.0426; the difference between both groups is therefore significant at the 5% level.

Five of the six patients tested positive for PD-L1 had an unresectable tumor at initial diagnosis. This feature is thus over-represented in the group of PD-L1 positive compared to the other patients. Table 6 shows the distribution of the PD-L1 status with respect to the resectability in a contingency table. The *p*-value according to Fisher’s exact test is 0.0102 suggesting a significant difference between both groups.

## 3. Discussion

### 3.1. c-Met

An association between the receptor tyrosine kinase c-Met and the pathogenesis of pancreatic cancer has already been demonstrated in several studies [7,10,11]. Neuzillet et al. have shown that an increased expression of c-Met in pancreatic tumor tissue was accompanied by an increased recurrence rate and a shorter survival time [12]. Furthermore, another study suggested that high expression of MET-mRNA is associated with a poorer prognosis [13]. However, both investigations required a surgical resection preparation. A method that determines the c-Met expression status by means of a “liquid biopsy” solely based on a blood sample of the patient is not yet established.

Based on our experiment with pancreatic cell lines, we were able to demonstrate that some pancreatic carcinoma cell lines release exosomes to their cell medium which express the protein c-Met. The difference between the mean of all carcinoma cell lines tested and the benign HPDE6 cell line was significant (*p* = 0.013).

In addition, despite the rather small sample size, a significant difference was found between serum samples from patients with pancreatic carcinoma and those with a benign differential diagnosis (*p* < 0.001).

After identifying the optimal cutoff of >495, a diagnostic test based on c-Met resulted in a sensitivity of 70%, a specificity of 85% and a diagnostic odds ratio of 13:2. Sensitivity and specificity of the only clinically established tumor marker for pancreatic carcinoma, CA 19-9, are 78.2% and 82.8%, respectively, according to a meta-analysis by Poruk et al. [14]. The c-Met diagnostic test achieves a similar level of accuracy to CA 19-9 but is not significantly superior to the established test. Using a combination of c-Met and CA 19-9, the test specificity can be increased up to 89.5% without diminishing the sensitivity.

Since some patients with chronic pancreatitis showed increased fluorescence values, it can be assumed that the selectivity between PDAC and healthy normal population would be even higher, and the test would potentially have even better quality criteria if the comparison collective was chosen differently. However, since the challenge in clinical practice is to distinguish PDAC from other relevant differential diagnoses, the chosen reference collective seems to be more realistic.

In order to examine c-Met as a potential prognostic factor, another cutoff optimization was performed, leading to a twice as high threshold of >990. This might suggest that a strong expression of c-Met on exosomes is necessary to have prognostic influence. Whether the high c-Met expression speaks for a particularly aggressive tumor with a high tendency to metastasis, or whether the high c-Met expression is simply the result of an already advanced tumor growth with resulting large tumor mass remains unclear. Using Kaplan–Meier survival time analysis, the median survival time was 21.7 months for 20 patients with a maximum fluorescence intensity of 990 and 9.5 months for the 10 patients with a fluorescence intensity greater than 990, which is a significant difference according to the log-rank test (*p* < 0.001).

### 3.2. PD-L1

The mechanism of immune evasion is known to contribute to the progression of numerous tumor entities, including pancreatic cancer [9]. Several studies suggested that PD-L1 expression in pancreatic cancer is associated with a poor prognosis [15,16,17]. Again, the decisive advantage of our approach using circulating exosomes compared to previous studies is its non-invasiveness. 

Considering the results of PD-L1 expression on exosomes from cell culture media, the large variability of the individual values is noticeable. Some cell lines show highly positive results, while in others PD-L1 expression seems to be absent. Due to the high variability of the individual values and the consequently broad 95% confidence interval, the difference of the mean of all malign cell lines to HPDE6 is not significant.

Examining PD-L1 expression on exosomes from serum samples, the mean fluorescence of CP-patients is even higher than in PDAC-patients. Accordingly, PD-L1 is not suitable as a diagnostic marker for pancreatic cancer.

The question remains whether patients with positive PD-L1 status distinguish themselves from PD-L1 negative patients with a poorer prognosis. Using the optimized cutoff of >299 for this purpose and applying Kaplan–Meier analysis for both groups, the PD-L1 positive patients do have a significantly shorter postoperative survival time of 7.8 months compared with PD-L1 negative patients (17.2 months, *p* = 0.043 according to log-rank test). PD-L1 expression on exosomes can thus be considered as a negative prognostic factor for pancreatic cancer. Since exosomes are able to transmit signals in the organism over long distances [18], the question arises whether pancreatic carcinomas take advantage of PD-L1 expression on exosomes in order to escape the immune response. However, further research is needed to prove this presumption.

Another promising application of the presented method is the non-invasive performance of a predictive test. Currently, the clinical significance of checkpoint inhibitors, which use PD-1 and PD-L1 as their targets, is steadily increasing. Pancreatic cancer has so far hardly responded to monotherapy with checkpoint inhibitors [19]. The combination of several therapeutic strategies and a targeted selection of patients to be treated using predictive markers is expected to overcome the resistance of pancreatic cancer to immunotherapy [20]. A serologic marker that provides reliable information about the PD-L1 expression status of a tumor would greatly improve this situation.

## 4. Materials and Methods 

### 4.1. Cell Culture Media

An overview of the applied cell lines and the corresponding origin and cell media is shown in Table 7. All cell lines were grown in monolayer culture in a humidified atmosphere containing 5% CO_2_ at 37 °C. The used culture medium was withdrawn from the culture after three days of breeding, bottled and stored at 4 °C.

### 4.2. Patient Sera Collection and Processing 

Written informed consent (approved by the Ethikkommission Dresden on the 06.03.2007 EK 5903 2007) was obtained from all patients to include serum samples, which was taken from patients undergoing surgery of the pancreas straight before operation. After a rest period of 30 min, the samples were centrifuged for 10 min at 1500× *g*. The serum was then portioned into cryotubes of 500 μL each, which were stored at −75 °C until further use. The exact diagnosis of the patients was made histopathologically based on the surgical resection preparation.

### 4.3. Exosome Isolation 

For exosome isolation from cell culture medium, 2 mL of the sample were centrifuged at 2000× *g* for 30 min to clean the medium of cell debris. The supernatant was mixed with 1 mL of the Invitrogen Total Exosome Isolation Reagent (from cell culture media) and incubated overnight at 4 °C. After subsequent centrifugation at 10,000× *g* for 60 min, the supernatant was discarded, and the visible pellet was resuspended in 100 µL DPBS. For exosome isolation from serum, 250 µL of the sample were centrifuged at 10,000× *g* for 30 min. The supernatant was mixed with 40 µL of the Invitrogen Total Exosome Isolation Reagent (from serum) and incubated for 30 min at 4 °C. After subsequent centrifugation at 10,000× *g* for 10 min, the supernatant was discarded and the visible pellet was resuspended in 100 µL Dulbecco’s Phosphate Buffered Saline. 

### 4.4. Exosome Staining and Flow Cytometry Analysis

The isolated exosomes were admixed with 10 μL latex beads solution (by ThermoFisher Scientific, Darmstadt, Germany, Aldehyde/Sulfate surface, 4% *w*/*v*, 4 µm) and incubated for 15 min at room temperature with continuous rotation. Afterwards, the solution was mixed with 700 μL of DPBS and incubated again for 30 min. Subsequently, 100 μL of 10% bovine serum albumin (BSA) and 100 µl of 3 M glycin solutions were added as blocking reagents, followed by another 30 min of incubation. The samples were centrifuged for 3 min at 12,000× *g*, the supernatant was discarded, and the pellets dissolved in 40 µL of working medium (2% BSA). Each sample was now divided into two parts of 20 μL each. These parts formed the negative and positive control of each sample. A total of 5 μL of the diluted antibody (c-Met RRID: AB_10858224, dilution 1:3 or PD-L1 RRID:AB_940372, dilution 1:7) were used. The negative control was amended with 5 μL of DPBS instead. The mixture was incubated for 30 min at 4 °C with continuous rotation and then centrifuged for 3 min at 12,000× *g*. The supernatant was discarded, and the pellets dissolved in 40 µL of working medium each. A total of 3 µL of the secondary antibody (Alexa Fluor 488 Goat anti-Rabbit RID: AB_10694704 for samples with c-Met or Alexa Fluor 488 Goat anti-Mouse RRID: AB_1904025 for samples with PD-L1, each in 1:19 dilution) were added to all samples including the negative controls. Incubation and centrifugation were analogous to the procedure when using the primary antibody. The resulting pellets were washed three times by dissolving in 200 µL working medium and subsequent centrifugation with discard of the supernatant. The washed pellets were ready for analysis by flow cytometry after dissolving in 1 mL DPBS. The applied flow cytometer was LSR II by Becton and Dickinson. The detector voltages were determined as follows: front scatter—500 V, side scatter—300 V, Alexa Fluor 488—520 V. The number of individual particles to be analyzed per sample was limited to 20,000. The target value was defined as the fluorescence difference between positive and negative controls of each sample.

### 4.5. Statistical Analysis

Statistical analysis was performed using Microsoft Excel 2016 and R embedded in RStudio. The confidence interval calculator [23] was used to calculate the confidence intervals of sensitivities, specificities and odds ratios. To detect significant differences in fluorescence intensity between two groups, the two-sided Mann–Whitney U test was used as a non-parametric test, since normal distribution of the populations was not given. Differences in the frequencies of positive and negative test results in different groups were determined by chi-squared homogeneity test or, in case of very low total numbers, by Fisher’s exact test. Cutoff optimization of the test procedures was performed using ROC curves and Youden indexes. In the case of the combined ROC curve of c-met and CA 19-9, we performed a method introduced by Haker et al. [24]. Survival time analyzes were performed using the Kaplan–Meier method. To determine significant differences in the survival time of two groups, the log-rank test according to Mantel–Cox was used.

## 5. Conclusions

c-Met is overexpressed on exosomes deriving from pancreatic cancer cells. The detection of c-Met positive exosomes in peripheral blood can be used as a diagnostic test with a similar level of accuracy as the only clinically established biomarker for pancreatic cancer, CA 19-9. A combined panel with both markers leads to an even improved test accuracy. Furthermore, the detection of c-Met positive exosomes in PDAC-patients is accompanied with a poorer prognosis.

PD-L1 is not proven to be overexpressed on exosomes deriving from pancreatic cancer cells; it therefore has no diagnostic value. The detection of PD-L1 positive exosomes in PDAC-patients, however, is accompanied with a poorer prognosis, as well. Another potential benefit of the described method is its use as a predictive test for oncotherapy with checkpoint inhibitors.

Further research will be necessary to confirm the findings displayed above in prospective studies on larger patient cohorts.

## Figures and Tables

**Table 1:** Diagnosis, gender and age of the analyzed patient population.

Variable	PDAC			SCA			CP		
	Total	c-Met	PD-L1	Total	c-Met	PD-L1	Total	c-Met	PD-L1
Total	55	30	36	10	10	0	24	10	17
Male	52.7%	53.3%	52.8%	30%	30%	-	70.8%	90%	64.7%
Female	47.3%	46.7%	47.2%	70%	70%	-	29.2%	10%	35.3%
Median Age (yrs.)	69	66.5	70.5	73	73	-	51	48	53
Mean Age (yrs.)	66.7	66.2	68.4	68.4	68.4	-	50.5	49.4	51.5

c-Met: Proto-oncogene Mesenchymal-epithelial Transition Factor; PD-L1: Programmed Cell Death 1 Ligand 1; SCA: Serous Cyst Adenoma; CP: Chronic Pancreatitis; PDAC: Pancreatic Ductal Adenocarcinoma.

**Table 2:** TNM stages and treatment intentions of analyzed PDAC-patients.

Variable	T		N		M		Intention	
	T_1–2_	T_3–4_	N_0_	N_pos_	M_0_	M_pos_	Curative	Unresectable
Total	1.8%	83.6%	20%	58.2%	78.2%	14.5%	79.2%	21.8%
c-Met	3.3%	90%	23.3%	70%	93.3%	6.7%	93.3%	6.7%
PD-L1	0%	77.8%	22.2%	44.4%	66.7%	22.2%	66.7%	33.3%

Difference to 100% due to unknown stages (T_X_, N_X_, M_X_).

**Table 3:** Mean estimators and 95% confidence interval of the various entities using the primary antibody anti-c-Met. *p*-value is based on Mann–Whitney U test. *** significant at 0.1% level (*p* < 0.001).

	PDAC	Non-malignant	SCA	CP
Lower Endpoint	629.2	175.7	45.4	153.8
Mean Estimator	889.4	356.8	228.8	487.7
Upper Endpoint	1149.6	537.8	412.2	815.6
	*** *p* < 0.001			

**Table 4:** Mean estimators and 95% confidence interval of the various entities using the primary antibody anti-PD-L1.

	PDAC	CP
Lower Endpoint	107.4	90.1
Mean Estimator	233.1	287.0
Upper Endpoint	358.9	483.9

**Table 5:** Comparison of sensitivity, specificity and odds ratio of diagnostic test based on c-Met and CA 19-9 alone or in combination.

Cut-Off Value	Sensitivity	Specificity	Odds Ratio
	(95% CI)	(95% CI)	(95% CI)
c-Met >495	70%	85%	13.2
	(52.1%–83.3%)	(64.0%–94.8%)	(3.1–56.6)
CA 19-9 >37 U/mL	72.4%	84.2%	14.0
	(54.3%–85.3%)	(54.3%–94.5%)	(3.2–61.4)
c-Met >543 or CA 19-9 >97 U/mL	72.4%	89.5%	22.2
	(54.3%–85.3%)	(68.6%–97.1%)	(4.2–119.3)

**Table 6:** Contingency table of the absolute number of patients with the features PD-L1 status and resectability. *p*-value is based on Fisher’s exact test. * Significant at 5% level (*p* < 0.05).

	Resectable	Unresectable
PD-L1 pos.	1	5
PD-L1 neg.	23	7
	* *p* = 0.0102	

**Table 7:** Overview of used cell lines, their origin and culture media.

Cell Line	Original Tissue	Provider	Cell Medium	Supplements
AsPc1	ascites metastases	ATCC	RPMI1640	10% FBS 10 mM HEPES 1 mM sodium pyruvate 4.5 g/l Glucose
BxPc-3	primary tumor			
HPDE6-E6E7c7	benign tissue	Ming-Sound Tsao, Toronto	KSFM	10% FBS
MiaPaCa-2	primary tumor	ATCC	DMEM	10% FBS 2.5% horse serum
PaCaDD 119	primary tumor	Surgical Laboratory, University Hospital Dresden [21,22]	2/3 DMEM 1/3 KSFM	20% FBS 1:100 Antibiotic/-mycotic
PaCaDD 137	primary tumor			
PaCaDD 161	liver metastases			
PaCaDD 165	ascites metastases			
PaCaDD 183	ascites metastases			
Panc-1	primary tumor	ATCC	RPMI1640	10% FBS
Su.86.88	liver metastases			

ATCC: American Type Culture Collection; DMEM: Dulbecco’s Modified Eagle’s Medium; HEPES: 4-(2-Hydroxyethyl)-1-Piperazineethanesulfonic Acid; KSFM: Keratinocyte Serum-free Medium; RPMI: Roswell Park Memorial Institute.

## Abbreviations

ATCC	American Type Culture Collection
AUC	Area Under the Curve
BSA	Bovine Serum Albumin
CA 19-9	Carbohydrate Antigen 19-9
c-Met	Proto-oncogene Mesenchymal-epithelial Transition Factor
CP	Chronic Pancreatitis
DMEM	Dulbecco’s Modified Eagle’s Medium
DPBS	Dulbecco’s Phosphate Buffered Saline
EV	Extracellular Vesicles
FBS	Fetal Bovine Serum
HEPES	4-(2-Hydroxyethyl)-1-Piperazineethanesulfonic Acid
HGFR	Hepatocyte Growth Factor Receptor
HPDE	Human Pancreatic Duct Epithelial Cell Line
KSFM	Keratinocyte Serum-free Medium
MAPK	Mitogen-activated Protein Kinase
(m)RNA	(Messenger) Ribonucleic Acid
NF-κB	Nuclear Factor “Kappa Light Chain Enhancer” of Activated B-Cells
PDAC	Pancreatic Ductal Adenocarcinoma
PD-1	Programmed Cell Death Protein 1
PD-L1	Programmed Cell Death 1 Ligand 1
PI3K	Phosphoinositide-3-Kinase
ROC	Receiver Operator Characteristics
RPMI	Roswell Park Memorial Institute
SCA	Serous Cyst Adenoma
STAT	Signal Transducers and Activators of Transcription
